# Genome-Wide Analysis of BnaRLCK VII Gene Family in *Brassica napus* and Investigation of Its Function in Resistance to *Sclerotinia sclerotiorum*

**DOI:** 10.3390/genes17070790

**Published:** 2026-07-12

**Authors:** Zining Guo, Zhuo Chen, Zheng Wang

**Affiliations:** School of Biological Science and Technology, Jiangsu University, Zhenjiang 212013, China; 2212317016@stmail.ujs.edu.cn (Z.G.); 222217015@stmail.ujs.edu.cn (Z.C.)

**Keywords:** *Brassica napus*, *BnaRLCK VII* gene family, *S. sclerotiorum*, *BnaBIK1*

## Abstract

Receptor-like cytoplasmic kinases (RLCKs) constitute a core family of signaling proteins that modulate diverse cellular activities and participate in multiple physiological and biochemical processes in plants. As a unique subclade of RLCKs, the receptor-like cytoplasmic kinases VII (RLCK VII) subfamily features a conserved kinase domain but lacks extracellular and transmembrane domains, and has been proven to exert crucial functions in plant immunity, growth and development, and yield formation. Background/Objectives: This study systematically identified and characterized RLCK VII family genes in the rapeseed (*Brassica napus*) genome, and explored their biological functions in plant resistance to *Sclerotinia sclerotiorum*. Methods: Multiple bioinformatics databases and online tools were employed to conduct a comprehensive analysis of the identified BnaRLCK VII genes, including their physicochemical properties, signal peptide characteristics, subcellular localization, phylogenetic relationships, gene structure, conserved motifs and domains, *cis*-regulatory elements, tissue-specific expression patterns, and gene duplication events. Transcriptome profiling (RNA-seq) and transient overexpression assays in *Nicotiana tabacum* were further performed to verify the function of BnaRLCK VII genes in *S. sclerotiorum* resistance. Results: A total of 173 RLCK VII family members were identified from the *B. napus* genome and designated as BnaRLCK VII genes. These genes were clustered into 10 distinct subgroups based on phylogenetic analysis. We further comprehensively analyzed their gene structures, conserved motifs, cis-acting elements, duplication patterns, and tissue expression profiles. RNA-seq analysis revealed that 31 BnaRLCK VII genes were significantly differentially expressed following *S. sclerotiorum* infection. Among these differentially expressed genes (DEGs), a homolog of *Arabidopsis thaliana* BIK1 was selected for functional validation and named *BnaBIK1*. Agrobacterium-mediated transient infiltration assays on tobacco leaves demonstrated that overexpression of BnaBIK1 could improve plant tolerance to *S. sclerotiorum*, which indicates that *BnaBIK1* is a promising candidate gene for regulating rapeseed resistance against this pathogen. Conclusions: Collectively, these findings indicate that BnaRLCK VII family genes, particularly *BnaBIK1*, serve as key positive regulators of *S. sclerotiorum* resistance in *B. napus*.

## 1. Introduction

China’s annual consumption of vegetable oil reaches approximately 40 million tons. Nevertheless, the country’s self-sufficiency rate for edible vegetable oil has long remained stable at roughly 30% [1], highlighting an urgent need to improve domestic edible vegetable oil production. Rapeseed (*Brassica napus* L.) is one of the most indispensable oil crops worldwide. In China, rapeseed oil accounts for nearly 50% of the total edible oil production from oil crops [2,3,4,5], making it a core source of domestic vegetable oil supply. To satisfy growing market and consumer demands, extensive research has focused on the genetic improvement and yield enhancement of rapeseed [6,7]. The Yangtze River Basin, the primary rapeseed-producing region in China, is severely plagued by rapeseed sclerotinia stem rot, a destructive fungal disease caused by *Sclerotinia sclerotiorum*. This pathogen causes a yearly yield loss of 10–20% in domestic rapeseed production [8]. Therefore, dissecting the molecular mechanism underlying rapeseed resistance to *S. sclerotiorum* and breeding resistant rapeseed varieties are crucial for safeguarding sustainable rapeseed production in China [9].

Receptor-like kinases (RLKs), first identified in *Zea mays* L., share structural homology with animal receptor tyrosine kinases [10,11]. RLKs are cell surface-localized receptor proteins defined by conserved intracellular kinase domains (KDs), including Pkinase-Tyr (pfam07714) and Pkinase (pfam00069). Most RLKs possess a typical modular structure consisting of a transmembrane (TM) domain and an extracellular ligand-binding domain (ECD), such as leucine-rich repeat (LRR), wall-associated kinase (WAK)-like, and lectin domains [12,13]. RLK members that lack extracellular domains are classified as the receptor-like cytoplasmic kinase (RLCK) subfamily [12]. Functionally, RLCKs primarily act as downstream signaling components of pattern recognition receptors (PRRs), transducing extracellular environmental and immune signals to trigger intracellular responses. They participate extensively in diverse plant physiological processes, including biotic and abiotic stress tolerance, as well as plant growth and development [14,15].

Early classification divided RLCKs into the TAK-like subfamily and RLCK I–XI subfamilies based on the conserved features of kinase subdomains and the presence or absence of transmembrane domains [12,16]. Subsequent phylogenetic analyses of RLK sequences from *Arabidopsis thaliana* and rice further reclassified plant RLCKs into 17 subfamilies, designated RLCK II and RLCK IV–XIX [17]. Among all RLCK subfamilies, RLCK VII is one of the most well-characterized subgroups, with genome-wide identification and functional analysis conducted in multiple plant species. To date, 46 RLCK VII members have been identified in *Arabidopsis* and 129 in upland cotton (*Gossypium hirsutum*), with several genes functionally validated [13,14,18,19]. However, the molecular diversity and biological functions of RLCK VII members remain largely unexplored in numerous other plant species.

Previous studies have verified that the RLCK VII subfamily plays essential roles in plant immunity, growth, and development [14,18]. Plant innate immunity is primarily composed of two layered defense responses: pathogen-associated molecular pattern (PAMP)-triggered immunity (PTI) and effector-triggered immunity (ETI). Accumulating evidence indicates that RLCK VII members serve as pivotal regulators involved in both PTI and ETI signaling pathways. In rice, the silencing of *OsRLCK57*, *OsRLCK107*, and *OsRLCK118* represses chitin- and peptidoglycan (PGN)-induced reactive oxygen species (ROS) accumulation and the expression of defense-related gene [20]. In *Arabidopsis*, BR-SIGNALING KINASE 1 (BSK1) physically interacts with FLAGELLIN SENSING 2 (FLS2) to mediate FLS2-dependent ROS bursts during immune activation [21]. As a central immune kinase, BOTRYTIS-INDUCED KINASE 1 (BIK1) contributes to broad-spectrum pathogen resistance by integrating multiple plant immune signaling pathways, promoting ROS generation, and modulating the transcription of defense genes in *Arabidopsis* [22,23,24,25]. In addition, the *Arabidopsis* kinase PBS1 (AvrPphB SUSCEPTIBLE 1) can be cleaved by the *Pseudomonas syringae* effector AvrPphB, thereby activating RPS5 (RESISTANCE TO PSEUDOMONAS SYRINGAE 5)-mediated ETI. Several PBS1-like (PBL) kinases, including BIK1, PBL1, and PBL2, are also targeted and cleaved by AvrPphB, which further suppresses PTI signaling [26,27,28]. PBL27 relays immune signals to the mitogen-activated protein kinase (MAPK) cascade via physical association with MAPKKK5 (MITOGEN-ACTIVATED PROTEIN KINASE KINASE KINASE 5) [29]. Both PBL2 and RIPK (RECEPTOR-INTERACTING CYTOPLASMIC KINASE) are critical for *XopAC*-triggered immunity against *Xanthomonas campestris pv. campestris* (*Xcc*) in *Arabidopsis* [30]. Furthermore, PBL2 and BSK family proteins participate in the recognition of the pathogen effector AvrAC and mediate downstream ETI activation [31]. Considering that RLCK VII members confer plant resistance to fungal pathogens such as *Botrytis cinerea*—a hemibiotrophic fungus with similar infection characteristics to *S. sclerotiorum*—functional characterization of RLCK VII genes in *B. napus* could provide valuable insights into the molecular mechanisms underlying rapeseed resistance to *S. sclerotiorum*. BnaRLCKVII proteins function as core cytoplasmic signaling kinases downstream of immune pattern recognition receptors; upon recognition of pathogen molecular signals, they trigger ROS burst, activate JA, ABA and SA hormone signaling cascades, and upregulate downstream defense genes to suppress hyphal expansion of *S. sclerotiorum*, so systematic functional characterization of RLCK VII genes in *B. napus* could provide valuable insights into the molecular mechanisms underlying rapeseed resistance to this pathogen [32,33,34].

In the present study, a total of 173 RLCK VII family members were identified from the *B. napus* genome and designated as BnaRLCK VIIs. We systematically characterized their physicochemical properties, predicted their subcellular localization, and classified these genes into 10 subgroups based on phylogenetic analysis. A comprehensive set of bioinformatics analyses was further performed, including the investigation of conserved domains, gene structures, gene duplication events, collinearity relationships, *cis*-regulatory elements in gene promoters, and tissue-specific expression patterns. As core components of plant PTI signaling, *BnaRLCK VII* proteins act as critical signal transducers during the interaction between cruciferous plants and *S. sclerotiorum*. When *S. sclerotiorum* invades host cells and releases PAMPs such as chitin, membrane-localized pattern recognition receptors can recognize these molecular signals and subsequently phosphorylate *BnaRLCK VII* members, represented by *BnaBIK1*. The activated kinases further trigger a series of downstream immune responses: they initiate ROS burst to directly inhibit the growth and spread of fungal hyphae, induce calcium influx to amplify immune signals, and activate the MAPK cascade to regulate the expression of downstream transcription factors. These signaling events jointly modulate the salicylic acid, jasmonic acid and ethylene signaling pathways, reinforce cell wall structure to resist fungal penetration, and confine pathogen-induced lesions to a limited area. The functional redundancy and subgroup differentiation of the large *BnaRLCK VII* family also enable rapeseed to form a stable and multi-layered defense system against *S. sclerotiorum* infection. In addition, transcriptome profiling was conducted to explore the potential association of BnaRLCK VII genes with rapeseed resistance to *S. sclerotiorum*, and 31 differentially expressed genes (DEGs) were screened out. Among these DEGs, a gene homologous to *Arabidopsis AtBIK1* was selected for further functional validation and named *BnaBIK1*. Agrobacterium-mediated transient infiltration of tobacco leaves combined with pathogen inoculation assays revealed that *BnaBIK1* overexpression greatly strengthens plant defense against *S. sclerotiorum* infection. Collectively, this study provides novel insights and a fundamental basis for the future functional characterization of BnaRLCK VII family genes.

## 2. Materials and Methods

### 2.1. Identification and Classification of the RLCK VII Gene Family in B. napus

To identify RLCK VII members in *B. napus*, we obtained protein sequences of 46 *Arabidopsis* RLCK VIIs from TAIR (https://www.arabidopsis.org/) (accessed on 2 July 2026). Using these sequences as queries, we ran BLASTP on the BnIR platform (http://yanglab.hzau.edu.cn/BnIR/BLAST) (accessed on 2 July 2026) to collect candidate RLCK VII genes in *B. napus*. Conserved domain models of Pkinase (pfam00069) and Pkinase-Tyr (pfam07714) were downloaded from Pfam (https://pfam.xfam.org), and HMMER 3.0 was used to screen proteins containing both domains. After removing redundant sequences, we rechecked the domain composition of candidates via SMART (http://smart.embl-heidelberg.de/) and NCBI CDD (https://www.ncbi.nlm.nih.gov/cdd) (accessed on 2 July 2026). Finally, proteins with Pkinase and Pkinase-Tyr domains but lacking transmembrane and extracellular domains were identified as RLCK VII members and named BnaRLCK VIIs [19,35,36,37,38,39].

### 2.2. Physicochemical Properties, Signal Peptides and Subcellular Localization Prediction of BnaRLCK VIIs

Furthermore, the physicochemical properties of candidate proteins, including theoretical isoelectric point (pI), molecular weight (MW), instability index, aliphatic index and grand average of hydropathicity (GRAVY), were calculated using the ExPASy ProtParam tool (https://web.expasy.org/protparam/) (accessed on 2 July 2026) [40]. Signal peptides and subcellular localization of BnaRLCK VII proteins were subsequently predicted via TargetP-2.0 (https://services.healthtech.dtu.dk/services/TargetP-2.0/) (accessed on 2 July 2026) and DeepLoc-2.1 (https://services.healthtech.dtu.dk/services/DeepLoc-2.1/) (accessed on 2 July 2026), respectively [41,42].

### 2.3. Phylogenetic Analysis of BnaRLCK VIIs

To classify members of the RLCK VII subfamily, phylogenetic analysis of BnaRLCK VII proteins was conducted using TBtools (v1.09) [43]. Protein sequences of 46 AtRLCK VIIs and BnaRLCK VIIs were aligned via the Muscle Wrapper and trimmed with the trimAL Wrapper. A maximum likelihood (ML) phylogenetic tree was then constructed using the IQ-tree Wrapper with 1000 bootstrap replicates [19]. The resulting tree was further edited on the Interactive Tree Of Life (iTOL) web server (https://itol.embl.de/itol.cgi) (accessed on 2 July 2026) [44].

### 2.4. Gene Structure, Conserved Motifs and Conserved Domain Analysis of BnaRLCK VIIs

Genome sequences, protein sequences and Generic Feature Format Version 3 (GFF3) files were downloaded from the BnIR database. To predict conserved motifs of BnaRLCK VII proteins, we submitted their protein sequences to the MEME Suite (https://meme-suite.org/meme/tools/meme) (accessed on 2 July 2026). We set the search to identify up to 18 conserved motifs with lengths ranging from 30 to 150 aa [45]. Conserved domain information of BnaRLCK VIIs was retrieved from the NCBINCBINCB and SMART databases. Finally, TBtools was used to visualize the gene structures, conserved motifs and conserved domains of these genes [43].

### 2.5. Chromosome Localization, Gene Duplication, and Collinearity Analysis of BnaRLCK VIIs

Chromosomal locations of BnaRLCK VII genes were extracted from the Generic Feature Format Version 3 (GFF3) files. Gene duplication events were analyzed via the all-vs-all BLAST module (http://yanglab.hzau.edu.cn/BnIR/BLAST) (accessed on 2 July 2026) embedded in TBtools. Based on the all-vs-all BLAST results, collinearity analysis of BnaRLCK VII members was conducted using the MCScanX module of TBtools. All analytical results were visualized with the Advanced Circos tool in TBtools [36].

The non-synonymous (Ka)/synonymous (Ks) substitution ratios, which reflect selection pressure, between different *B. napus* varieties were retrieved from the BnVIR database (https://yanglab.hzau.edu.cn/BnVIR) (accessed on 2 July 2026).

### 2.6. Promoter Analysis of BnaRLCK VIIs

The 2000 bp upstream genomic sequences of BnaRLCK VIIs were extracted as candidate promoters with TBtools. *Cis*-acting elements were predicted using PlantCARE (http://bioinformatics.psb.ugent.be/webtools/plantcare/html/) (accessed on 2 July 2026) [46]. TBtools was finally applied to visualize the distribution of *cis*-elements in the promoters of BnaRLCK VIIs [43].

### 2.7. Tissue Expression Profile of BnaRLCK VIIs

Transcript expression data of BnaRLCK VII genes in nine distinct rapeseed tissues, including 4 mm buds, filaments, petals, pollen, 14-day-old leaves, roots, 28-day-after-flowering (28DAF) seeds, 48-day-after-flowering (48DAF) siliques, and middle stem peels, were retrieved from the BnIR database [37]. All transcript data used in this analysis were downloaded from the public online BnIR database [37]; the original sequencing experiment deposited in this database set three biological replicates for leaf tissue, with multiple uniform plant leaves pooled per replicate to eliminate individual variation. Gene expression profiles were normalized using the log2(TPM+1) algorithm and visualized as heatmaps via the built-in heatmap tool in TBtools [6,43,47].

### 2.8. RNA-Seq

The transcriptome data used in this study were retrieved from a previously published public dataset concerning the responses of *B. napus* to *S. sclerotiorum* infection [48]. Raw reads were filtered to remove low-quality sequences, and the resulting clean reads were used for subsequent bioinformatics analysis [49,50]. The clean reads were aligned to the *B. napus* v4.1 reference genome using the STAR software (v2.7.10a) [51]. Gene expression levels were quantified as fragments per kilobase of transcript per million mapped reads (FPKM) using RSEM [52]. Differentially expressed genes (DEGs) were identified via DESeq2 (v1.26.0) with the threshold of adjusted *p*-value (padj) < 0.05 and |log2FoldChange| > 1 [53]. Finally, the expression patterns of BnaRLCK VII genes under *S. sclerotiorum* treatment were visualized in heatmaps using TBtools based on log2(FPKM+1) normalization [43].

### 2.9. RT-qPCR

For RT-qPCR validation, total RNA was isolated from *B. napus* leaves with or without *S. sclerotiorum* inoculation [54]. First-strand cDNA was reverse-transcribed using the EasyScript^®^ All-in-One First-Strand cDNA Synthesis SuperMix for qPCR (TransGen Biotech, Beijing, China). Quantitative PCR was conducted on the QuantStudio 3 PCR System (Applied Biosystems, Foster City, CA, USA) with PerfectStart^®^ Green qPCR SuperMix (TransGen Biotech, Beijing, China). All genes were analyzed in three biological replicates, with *BnaActin* employed as the internal reference gene. The 2^−ΔΔCt^ method was used for calculating the relative expression level of the target genes [55]. All primer sequences used in this study are listed in Table S4.

### 2.10. Transient Transformation of Nicotiana tabacum and Inoculation Experiment with S. sclerotiorum

In this study, the pCAMBIA1300 plasmid was used as the backbone vector to construct the *BnaBIK1* overexpression vector.

First, the pCAMBIA1300 vector was double-digested with restriction endonucleases *Kpn* I and *BamH* I in a total 50 μL reaction system. The mixture was incubated at 37 °C for 2 to 4 h. The digested products were separated by 1% agarose gel electrophoresis at 120 V for 40 min. The linearized vector was recovered using a gel extraction kit, and its nucleic acid concentration was determined with a micro-volume spectrophotometer.

Second, the previously constructed *BnaBIK1* cloning vector was used as the template. The full-length sequence of *BnaBIK1* was amplified by PCR with the primer pair BIK1-F/BIK1-R (Table S4), and homologous arms were subsequently added to the amplified fragment using primers vector-F/vector-R(Table S4). The 50 μL PCR reaction system was prepared with 2 × TransStrat FastPfu PCR SuperMix. The amplification program was set as follows: initial denaturation at 95 °C for 3 min; 34 cycles of 95 °C for 15 s, 58 °C for 20 s and 72 °C for 1 min; and a final extension step at 72 °C for 5 min. The PCR products were separated by 1% agarose gel electrophoresis (120 V, 400 mA) for 25 min, and the target fragment was purified.

Subsequently, homologous recombination between the linearized vector and purified target fragment was performed in a 10 μL reaction system using the pEASY^®^-Basic Seamless Cloning and Assembly Kit. The reaction mixture was incubated at 50 °C for 15 min and immediately cooled on ice upon completion.

The recombination product was transformed into competent *Escherichia coli* cells. Positive recombinants were screened via PCR identification using primers 35S-F and BIK1-FLAG-R, and further verified by Sanger sequencing. Finally, the sequence-verified recombinant plasmid *35S:BnaBIK1* was transformed into *Agrobacterium tumefaciens* for subsequent transient expression assays. [56]. Four-week-old tobacco seedlings were subjected to transient transformation, followed by inoculation with *S. sclerotiorum*. The fungal strain was activated and cultured on potato dextrose agar (PDA) medium at 22 °C in darkness for three days to obtain fresh and active mycelia. For uniform inoculation, 5 mm-diameter fungal plugs were excised from the growing edge of fungal colonies and placed on the center of fully expanded detached tobacco leaves at 12 h after transient transformation. All inoculated leaves were incubated in sterile Petri dishes with moist filter paper at 22 °C and 85% relative humidity under a 16 h light/8 h dark photoperiod. After 24 h of inoculation, leaf lesions were photographed at a fixed height, and the lesion area of each sample was accurately quantified using ImageJ software (V1.8.0345) [57].

### 2.11. Statistical Analysis

Statistical analysis was performed using GraphPad Prism 8 software. The *p* values was calculated by two-tailed *t*-test and the asterisks represent the significant difference (*: *p* < 0.05, **: *p* < 0.01, ***: *p* < 0.001, ****: *p* < 0.0001).

## 3. Results

### 3.1. Identification of BnaRLCK VIIs and Prediction of Their Physicochemical Properties, Signal Peptides, and Subcellular Localization

To identify RLCK VII family members in *B. napus*, protein sequences of 46 AtRLCK VII members were retrieved from the TAIR database and used as queries for BLASTP searches against the BnIR database. After eliminating redundant sequences, a total of 208 candidate proteins were retained. For further domain verification, two hidden Markov model (HMM) profiles corresponding to the Pkinase (pfam00069) and Pkinase-Tyr (pfam07714) domains were downloaded from the Pfam database and searched using HMMER 3.0. All 208 candidates were confirmed to harbor both conserved kinase domains. In total, 35 sequences were excluded due to the presence of transmembrane or extracellular domains, which are atypical characteristics of the RLCK VII subfamily [19]. Finally, a total of 173 proteins were confirmed as bona fide RLCK VII members in *B. napus* and designated BnaRLCK VIIs (Table 1).

To characterize the physicochemical properties of BnaRLCK VII proteins, key parameters were calculated using the ExPASy database (Table 1). The protein length of BnaRLCK VII members varied from 160 aa to 1295 aa, with an average length of 424 aa. Their molecular weight (MW) ranged from 18.01 kDa to 145.96 kDa, with a mean value of 47.21 kDa. Specifically, 81.5% of BnaRLCK VII proteins had MW values between 40 kDa and 60 kDa, while only two proteins were smaller than 20 kDa and four proteins exceeded 80 kDa. Instability index (II) analysis indicated that 60.69% of BnaRLCK VII proteins had an II value below 40, and 34.68% had II values ranging from 40 to 50, demonstrating that the majority of BnaRLCK VII proteins are structurally stable. Moreover, most BnaRLCK VII proteins exhibited high aliphatic index (AI) values, suggesting their relatively high thermal stability. Theoretical pI analysis revealed that 73.4% of BnaRLCK VIIs are basic proteins with pI values greater than 7. In addition, all BnaRLCK VII proteins exhibited negative grand average of hydropathicity (GRAVY) values, demonstrating their hydrophilic nature. Collectively, these physicochemical properties offer basic clues for understanding the structural properties of the BnaRLCK VII family proteins.

Signal peptide prediction and subcellular localization analysis further showed that none of the BnaRLCK VII proteins contained signal peptides. Consistent with the typical features of RLCK family members, nearly all BnaRLCK VII proteins were localized in the cytoplasm or cytoplasmic membrane. Only two proteins were predicted to localize in the nucleus and one in the mitochondrion (Table 1 and Table S1). These results further validate the reliability of our genome-wide identification of BnaRLCK VII genes.

### 3.2. Classification of BnaRLCK VIIs and Their Conserved Motifs and Gene Structure Analysis

**Figure 1:**
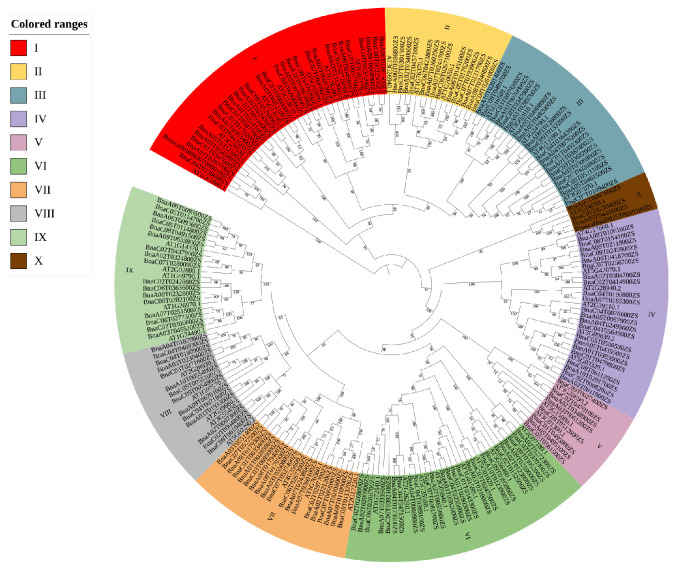
Phylogenetic tree depicting using the protein sequence of 173 BnaRLCK VIIs in *B. napus* and 46 AtRLCK VIIs in *A. thaliana*.

To clarify the phylogenetic relationships and classify the BnaRLCK VII members, a maximum likelihood (ML) phylogenetic tree was constructed in TBtools based on protein sequences of 46 AtRLCK VIIs and 173 BnaRLCK VIIs, with 1000 bootstrap replicates (Figure 1). A previous study has classified 46 *Arabidopsis* RLCK VII proteins into nine subgroups with three unclassified genes [18]. Following the classification criteria of AtRLCK VIIs, the BnaRLCK VII family was further divided into ten subgroups in this study.

To verify and refine the subgroup classification, we systematically analyzed the conserved motifs, conserved domains, and gene structures of all *BnaRLCK VII* members.

**Figure 2:**
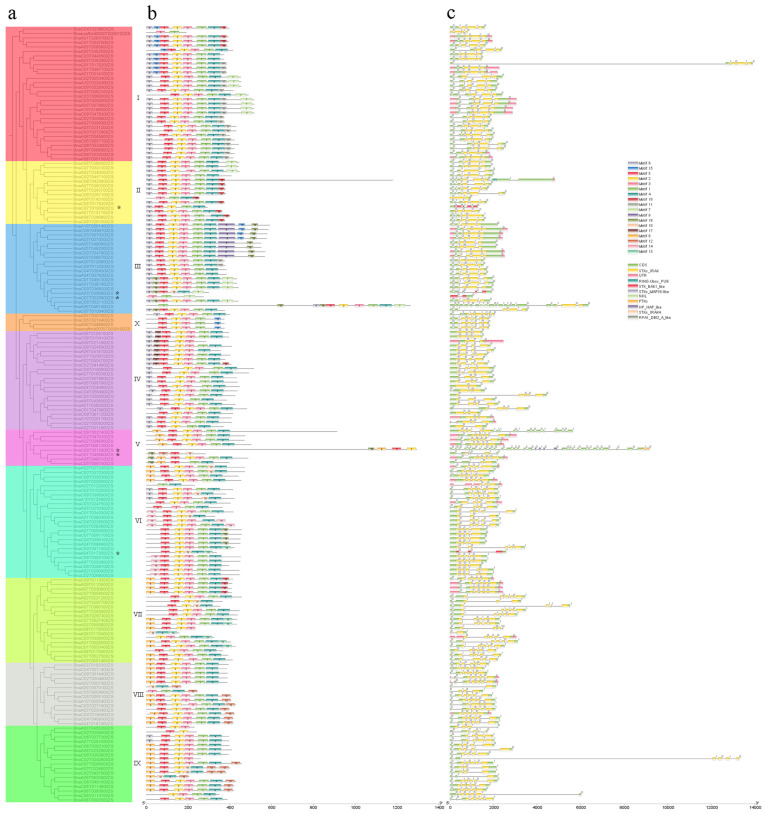
Phylogenetic relationship, conserved motifs, gene structure, and conserved domains of the BnaRLCK VII family. (**a**) Phylogenetic tree constructed from full-length protein sequences of 173 *BnaRLCK VII* genes, grouped into nine distinct subgroups (I–IX) with different background colors. (**b**) Conserved motif distribution in *BnaRLCK VII* proteins. Motifs numbered 1–18 were automatically identified and sorted by MEME software according to ascending E-value (lower E-value indicates higher sequence conservation). Each colored box represents one independent motif. Motif IDs are clearly labeled next to each color patch to avoid confusion caused by similar hues. (**c**) Gene structure and conserved domain architectures. UTR (untranslated region), CDS (coding sequence), and conserved domains are shown in different colors. Note that the phylogenetic tree in (**a**) was built using protein sequences, while panel (**c**) displays genomic exon-intron structures. Two long genes in subgroups I and IX show similar protein length but distinct intron-exon patterns within their own clades. Due to the limited color palette used in plotting, direct text labels are placed on each colored segment to distinguish visually similar elements: STKc-IRAK4 and PTKc domains are explicitly tagged, and CDS/UTR regions are clearly marked. Genes with atypical kinase domains (STK_BAK1_like, STKc_MAP3K-like, STKc_IRAK4) are marked with asterisks for easy identification.

First, a maximum-likelihood phylogenetic tree was constructed based on full-length protein sequences (Figure 2a). Subgroup-specific conserved motif patterns were observed in this tree.

Second, MEME-based conserved motif analysis (Figure 2b) showed that all *BnaRLCK VII* proteins carry complete or partial sequences of motifs 1–5. These five core motifs fully correspond to the PKc_like catalytic domain region, reflecting high sequence conservation across the whole family.

Third, we analyzed conserved domain architectures and gene exon-intron organizations (Figure 2c). Domain annotation indicated that all *BnaRLCK VII* proteins fall into the PKc_like superfamily. Most members contain the STKc_IRAK domain; four genes carry the STK_BAK1_like domain, three genes contain the STKc_MAP3K-like domain, another three harbor STKc_IRAK4, and several members have PTKc domains. Genes with atypical kinase domains are marked with asterisks in Figure 2c for easy identification. Gene structure statistics extracted from GFF3 files showed exon numbers ranging from 2 to 33, with 85.5% of the genes containing 4–6 exons. Three genes possess far more exons than others. Genes clustered within the same phylogenetic subgroup display highly consistent structural characteristics, including similar exon numbers and identical exon phasing patterns.

Collectively, the congruent patterns of conserved motifs, domain architectures and gene structures strongly support the reliability of our subgroup classification.

### 3.3. Chromosome Localization, Gene Duplication and Collinearity Analysis of BnaRLCK VIIs

**Figure 3:**
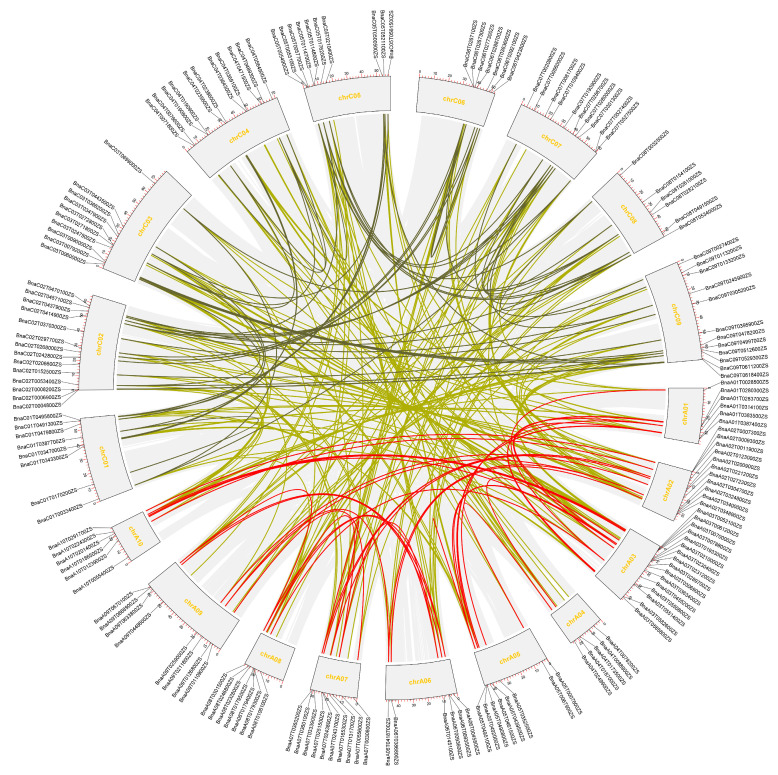
Collinearity analysis of *BnaRLCK VII* genes. The red lines represent the collinearity in the A sub-genome, the brown lines represent the collinearity in the C sub-genome, and the green lines represent the collinearity between the A sub-genome and the C sub-genome.

Chromosomal distribution information retrieved from the Generic Feature Format Version 3 (GFF3) files revealed that 83 BnaRLCK VII members are distributed across the A subgenome and 88 across the C subgenome. The remaining two genes, *Bnascaffold0025T0039100ZS* and *Bnascaffold0630T0000100ZS*, are located on unassembled scaffolds (Table 1, Figure 3). To explore the evolutionary relationships among BnaRLCK VII members, we characterized their duplication patterns and performed collinearity analysis (Table S2, Figure 3). Gene duplication events, including whole-genome duplication (WGD)/segmental duplication (SD), dispersed duplication (DSD), proximal duplication (PD) and tandem duplication (TD), are major driving forces for the evolution of gene families [58,59]. Our results showed that 159 BnaRLCK VII genes arose from WGD or segmental duplication. By comparison, 9 genes were derived from dispersed duplication, 2 from proximal duplication, and another 2 from tandem duplication (Table S2). These data indicate that WGD and segmental duplication are the predominant duplication modes during the evolution of the BnaRLCK VII family.

Collinearity analysis identified a total of 241 paralogous gene pairs from 171 BnaRLCK VII genes (the two scaffold-located genes were excluded from this analysis). Among these pairs, 38 were detected within the A subgenome, 53 within the C subgenome, and 150 between the A and C subgenomes (Figure 3), which is consistent with the gene duplication classification results (Table S2). Collectively, WGD and segmental duplication have played critical roles in the evolutionary expansion of the BnaRLCK VII gene family.

To evaluate the selection pressure on BnaRLCK VII genes, we retrieved their Ka/Ks ratios from the BnVIR database (Table S3). Only seven genes (*BnaA02G0272300ZS*, *BnaA06G0093600ZS*, *BnaC02G0152500ZS*, *BnaC02G0242800ZS*, *BnaC02G0414900ZS*, *BnaC04G0190900ZS* and *BnaC05G0114700ZS*) exhibited Ka/Ks values greater than 1. This finding demonstrates that most BnaRLCK VII genes have undergone strong purifying selection, which restricts functional divergence and maintains functional conservation throughout evolution [60].

### 3.4. Promoter Analysis of BnaRLCK VIIs

**Figure 4:**
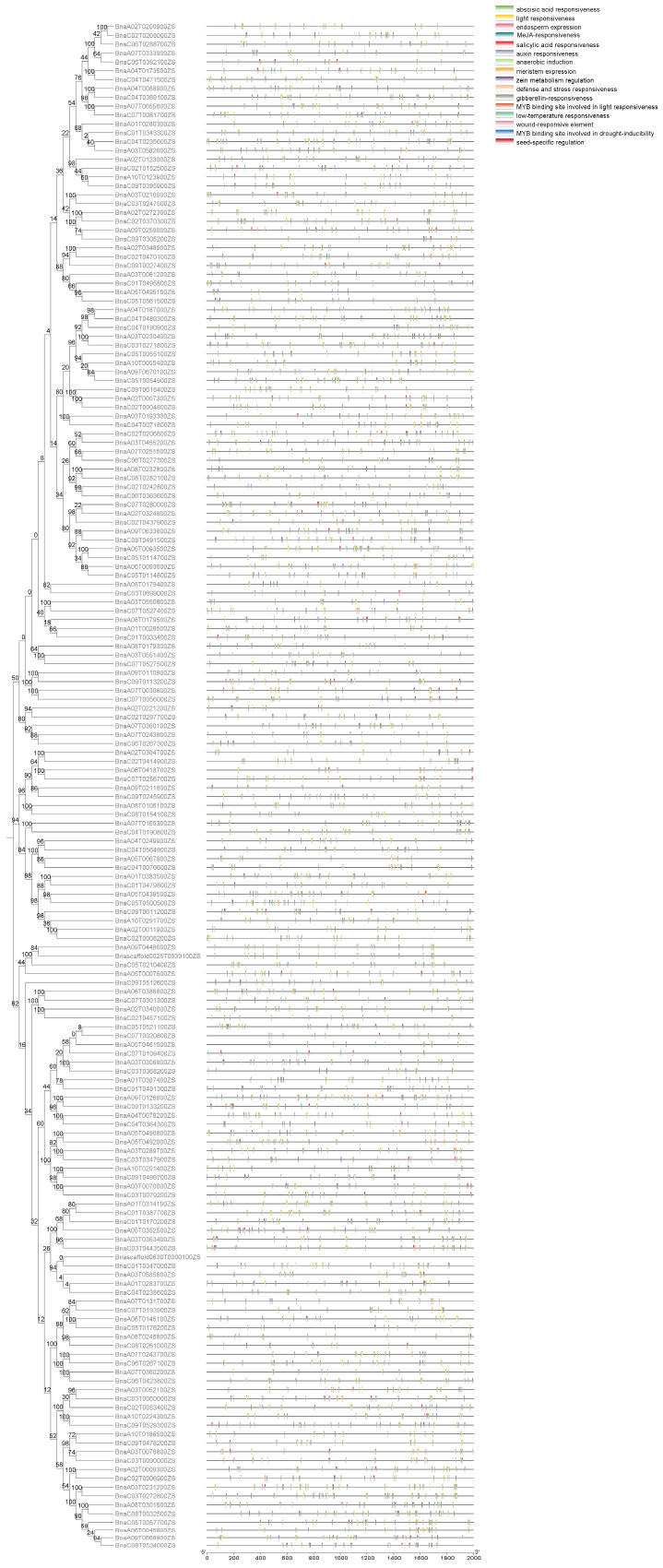
The prediction of *cis*-acting elements in *BnaRLCK VIIs* promoters. The *cis*-acting elements were divided into 16 classes based on their function.

To explore the transcriptional regulation of *BnaRLCK VII* genes, we analyzed their promoter sequences using the PlantCARE database. Due to the extremely short promoter length of *Bnascaffold0630T0000100ZS*, a total of 172 genes were retained for subsequent *cis*-element prediction. Apart from various stress- and hormone-related cis-elements, canonical core promoter elements including TATA-box and CAAT-box were widely detected in most promoter regions. Since TATA-box and CAAT-box serve as universal basal transcription initiation signals with limited functional specificity, we did not focus on them in the follow-up classification and visualization, and our analysis mainly centered on functional elements related to development and stress response. After screening and classification based on element functions, the predicted *cis*-acting elements were visualized via TBtools, and 16 categories of regulatory elements were identified (Figure 4). To further illustrate the frequency distribution of diverse functional *cis*-elements across individual promoters, a statistical heatmap was generated using TBtools (Figure S1).

Functional annotation showed that nearly all *BnaRLCK VII* genes harbor light-responsive elements, with the only exception of *BnaC07T0109400ZS*. In addition, 155 *BnaRLCK VII* members contain elements associated with anaerobic induction. A considerable number of genes carry hormone-responsive *cis*-elements, including 133 methyl jasmonate (MeJA)-responsive, 140 gibberellin (GA)-responsive, 92 abscisic acid (ABA)-responsive, and 80 salicylic acid (SA)-responsive genes (Figure S1). These results indicate that *BnaRLCK VII* genes are extensively involved in multiple hormone signaling pathways. Among hormone-associated motifs, MeJA- and ABA-responsive elements appeared at higher frequencies, suggesting MeJA and ABA signaling may act as primary regulatory modules mediating *BnaRLCK VII* biological functions. In addition, auxin-, endosperm-, seed-, zein metabolism- and meristem-specific cis-elements were detected in partial promoters, indicating that subsets of *BnaRLCK VII* genes potentially regulate plant growth and organ development. Rich stress-related cis-motifs also imply that several family members may respond to diverse abiotic stresses including drought and low temperature [61,62,63].

### 3.5. Tissue Expression Profile of BnaRLCK VIIs

**Figure 5:**
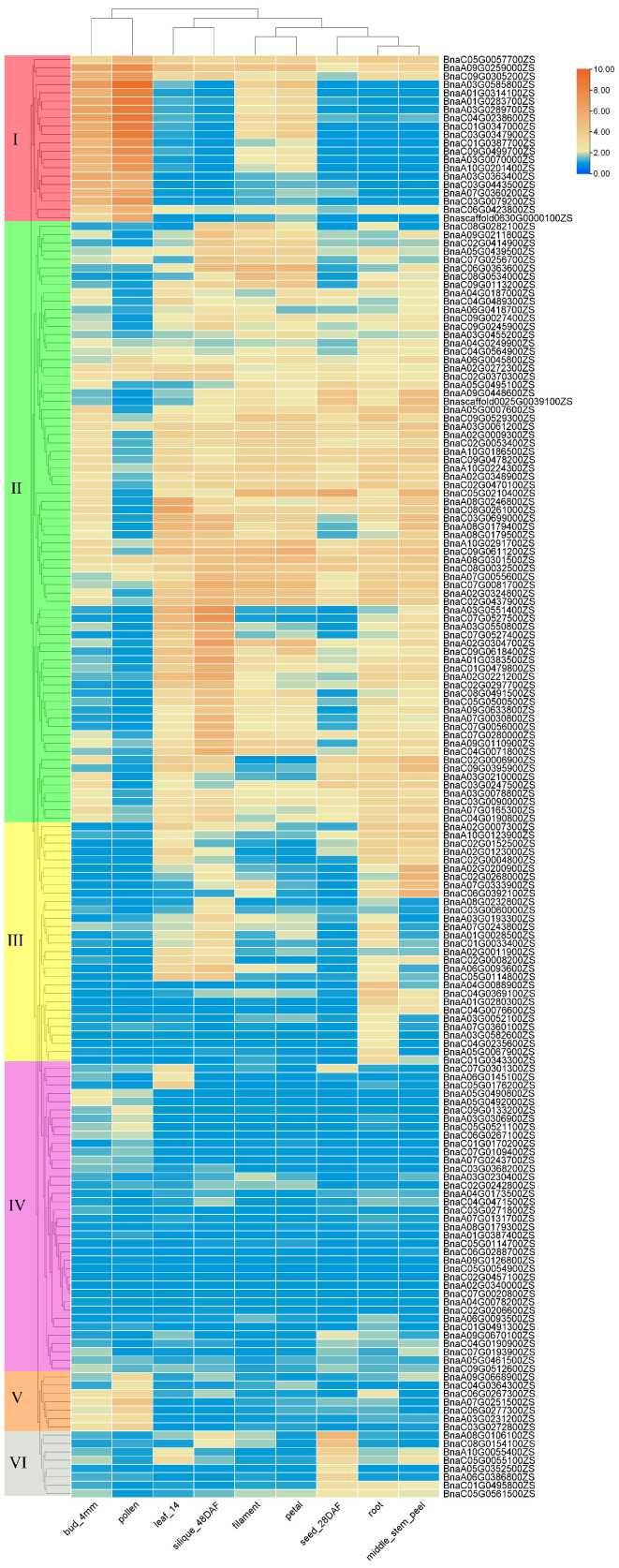
Tissue expression profile of *BnaRLCK VII* genes based on the RNA-seq data from BnIR database. A total nine tissues were selected. They were classified into 6 groups based on the cluster analysis.

Tissue-specific expression patterns are critical for inferring the potential biological functions of genes. Transcript expression data were collected from nine rapeseed tissues, including 4 mm buds, petals, filaments, pollen, roots, 14-day-old leaves, 28-day-after-flowering (28DAF) seeds, 48-day-after-flowering (48DAF) siliques, and middle stem peels. Expression clustering and visualization were performed using the heatmap tool in TBtools (Figure 5).

Based on expression clustering results, the *BnaRLCK VII* family was classified into six distinct subgroups. Group I genes exhibited extremely high expression levels in floral organs, particularly in flower buds and pollen, while Group V genes were specifically expressed in 4 mm buds and pollen, implying their essential roles in the reproductive development of *B. napus*. Group II genes were constitutively expressed across all detected tissues, indicating their widespread functions in plant growth and development. Group III genes were predominantly expressed in roots, and Group VI genes showed specific expression in 28DAF seeds, suggesting the potential involvement of Group III genes in root development and Group VI genes in seed development and maturation. Notably, Group IV genes were barely expressed in all examined tissues, which may indicate that these genes have no obvious function in vegetative and reproductive growth or have undergone functional loss during evolution. In general, most *BnaRLCK VII* genes exhibited distinct tissue-specific expression patterns, with only Group II genes showing ubiquitous expression (Figure 5).

### 3.6. Expression Analysis of BnaRLCK VIIs After the Treatment of S. sclerotiorum

**Figure 6:**
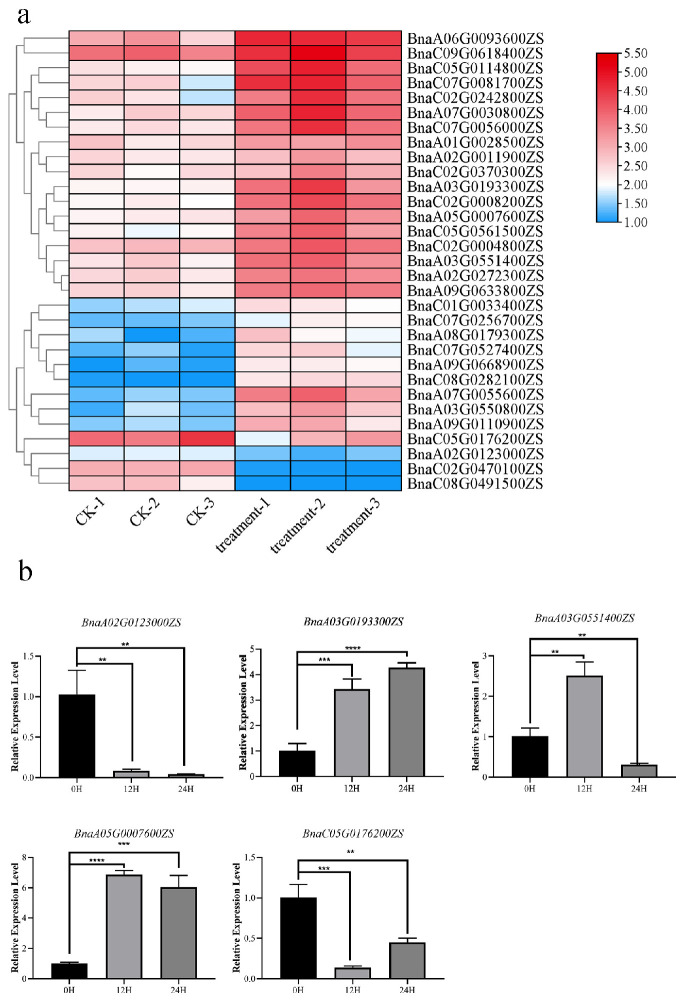
Changes in the expression levels of *BnaRLCK VII* genes under the treatment of *S. sclerotiorum*. (**a**) The differentially expressed genes (DEGs) of *BnaRLCK VIIs* after 36 h inoculation with *S. sclerotiorum*. (**b**) The RT-qPCR analysis of 5 *BnaRLCK VII* genes under different treatment durations. The asterisks indicate that the two-tailed *t*-test analysis shows a significant difference compared with the result of 0 H. (**: *p* < 0.01, ***: *p* < 0.001, ****: *p* < 0.0001).

To explore the transcriptional responses of *BnaRLCK VII* genes under *S. sclerotiorum* infection, transcriptome sequencing was performed to monitor their expression changes in *B. napus* after pathogen inoculation. Differential expression analysis revealed that a total of 31 *BnaRLCK VII* genes exhibited significant expression alterations upon *S. sclerotiorum* infection (Figure 6a). To further classify the induction levels of upregulated DEGs, gene expression changes were divided into three grades according to fold change (FC): low upregulation (1.5 ≤ FC < 2.0), moderate induction (2.0 ≤ FC < 4.0), and strong induction (FC ≥ 4.0). Among these differentially expressed genes (DEGs), 27 genes were upregulated, including 18 genes that were induced from moderate to high expression levels and nine genes that shifted from low to moderate expression levels. The remaining four DEGs were downregulated after inoculation. Among these downregulated genes, *BnaC05G0176200ZS* showed a slight reduction in expression after treatment despite its relatively high basal expression in uninoculated plants, while *BnaC08G0491500ZS* was silenced following *S. sclerotiorum* infection.

To validate the transcriptome results, five representative DEGs were selected for expression verification via RT-qPCR (Figure 6b). Consistent with the RNA-seq data, *BnaA02G0123000ZS* and *BnaC05G0176200ZS* were significantly downregulated, whereas *BnaA03G0193300ZS* and *BnaA05G0007600ZS* were upregulated after inoculation. Notably, *BnaA03G0551400ZS* displayed a distinct expression pattern, being significantly upregulated at 12 h post-inoculation but inhibited at 24 h post-inoculation.

### 3.7. Overexpression of BnaBIK1 Enhances the Resistance of Tobacco Against S. sclerotiorum

To further explore the biological functions of these disease-responsive DEGs in plant defense against *S. sclerotiorum* infection, the gene *BnaA03G0193300ZS*, a homologous gene of *Arabidopsis AtBIK1*, was selected for functional characterization and designated *BnaBIK1*. This candidate was prioritized based on previous studies demonstrating that *AtBIK1* positively regulates plant resistance to *B. cinerea*, a hemibiotrophic pathogen with a similar infection lifestyle to *S. sclerotiorum* [22,32].

To validate the transcriptome results, five representative DEGs were selected for expression verification via RT-qPCR (Figure 6b). Genes were chosen to cover distinct expression profiles identified in this study, including significantly downregulated genes, continuously upregulated genes, and a gene with a unique temporal expression pattern that was upregulated at 12 h but suppressed at 24 h post-inoculation. Consistent with the RNA-seq data, *BnaA02G0123000ZS* and *BnaC05G0176200ZS* were significantly downregulated, whereas *BnaA03G0193300ZS* and *BnaA05G0007600ZS* were upregulated after inoculation. Notably, *BnaA03G0551400ZS* displayed a distinct expression pattern, being significantly upregulated at 12 h post-inoculation but inhibited at 24 h post-inoculation.

**Figure 7:**
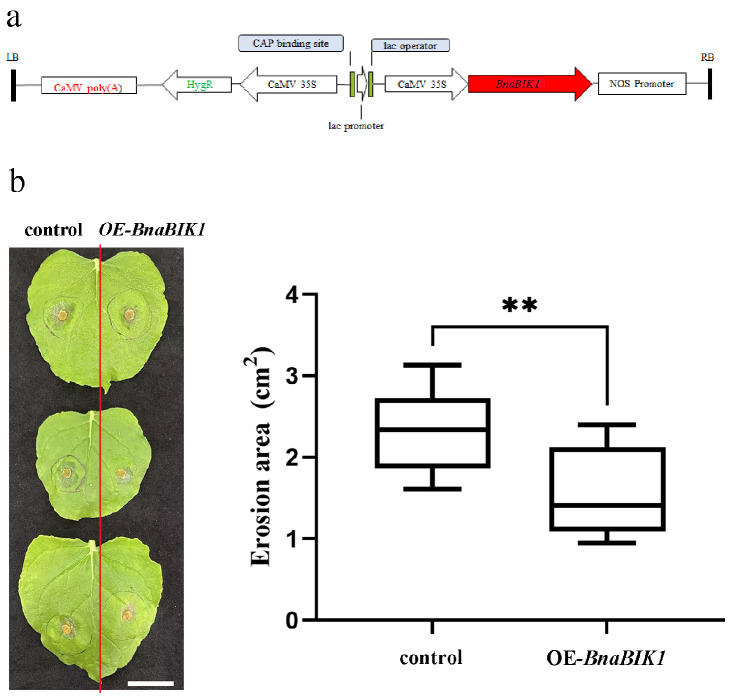
Pathogen inoculation assay after Agrobacterium-mediated transient infiltration of tobacco leaves with *BnaBIK1*. (**a**) The construction of *BnaBIK1* overexpression vector. (**b**) Statistics on the lesion area of *S. sclerotiorum* on tobacco leaves at 24 h after inoculation. Tobacco leaves were transiently transformed with the *BnaBIK1* overexpression vector or the empty vector 12 h in advance. The asterisks indicate that the two-tailed *t*-test analysis shows a significant difference compared with the control group. Scale bar = 3 cm. Error bars indicate SD (*n* = 10). (**: *p* < 0.01).

To investigate the role of *BnaBIK1* in plant defense against *S. sclerotiorum*, we introduced the recombinant vector into tobacco leaves via *Agrobacterium*-mediated transient infiltration, with leaves infiltrated with the empty vector serving as the negative control (Figure 7a). Twelve hours after Agrobacterium infiltration, the tobacco leaves were inoculated with *S. sclerotiorum*. Phenotypic observation revealed that the lesion area on *BnaBIK1*-overexpressing tobacco leaves was obviously smaller than that of the control group at 24 h post inoculation (Figure 7b). These results demonstrate that overexpression of BnaBIK1 markedly strengthens plant defense against infection by *S. sclerotiorum*.

## 4. Discussion

*B. napus* originated approximately 7500 years ago via interspecific hybridization between *Brassica rapa* and *Brassica oleracea*, followed by chromosome doubling, which represents a typical allopolyploidization event [64]. The modern diploid *Brassica* species, including *B. rapa* and *B. oleracea*, evolved from ancestral whole-genome triplication (WGT) and subsequent diploidization [64]. In the present study, a total of 173 *BnaRLCK VII* genes were identified in the *B. napus* genome. Each *Arabidopsis* RLCK VII member has 2–6 corresponding homologs in rapeseed, which is consistent with the polyploid evolutionary characteristics of *B. napus* (Figure 1).

Gene duplication events, including tandem duplication, transposition, whole-genome duplication (WGD), and segmental duplication, provide major genetic resources for the expansion and evolution of gene families [65]. Our gene duplication and collinearity analyses demonstrated that WGD and segmental duplication predominantly drove the expansion of the *BnaRLCK VII* family (Table S2, Figure 3). Collinearity results showed that more than 62.2% of paralogous gene pairs were distributed between the A and C subgenomes, indicating that WGD contributes more substantially than segmental duplication to the expansion of the *BnaRLCK VII* gene family.

The Ka/Ks ratio is a core indicator for evaluating selective pressure during gene evolution [66,67]. Ka/Ks analysis revealed that over 95.9% of *BnaRLCK VII* genes underwent strong purifying selection after family expansion. This evidence indicates that the *BnaRLCK VII* family is highly functionally conserved during evolution, implying their indispensable roles in maintaining normal growth and physiological activities of *B. napus* (Table S3) [68].

Physicochemical characterization and structural analysis showed that all 173 BnaRLCK VII proteins are hydrophilic and lack signal peptides. Subcellular localization prediction further confirmed that these proteins are mainly distributed in the cytoplasm or cytoplasmic membrane, supporting their functional roles in cytoplasmic signal transduction, which is consistent with the canonical functions of RLCK family proteins. In addition, conserved domain analysis identified several atypical functional domains in certain BnaRLCK VII members, including the HP_HAP_like histidine phosphatase domain, RING_Ubox domain, RPA1_DBD_A_like domain, and NHL domain. These diverse domain architectures suggest that individual *BnaRLCK VII* genes may participate in multiple physiological regulatory processes in plants, such as phosphate metabolism and protein degradation (Figure 2) [69,70].

Cis-acting element prediction demonstrated that *BnaRLCK VII* genes harbor multiple hormone-responsive elements associated with MeJA, GA, ABA, SA, and auxin signaling, implying their potential roles in regulating plant growth, development, and immune responses (Figure 4). Tissue expression profiles further validated the functional diversity of *BnaRLCK VII* genes, as several members exhibited tissue-specific expression patterns related to organ development (Figure 5). Sixteen subgroup I genes were specifically and highly expressed in buds, filaments, petals, and pollen, suggesting their indispensable functions in floral organ morphogenesis. Six subgroup III genes displayed root-specific expression with negligible transcription in other tissues, indicating their potential involvement in root development, nutrient uptake, and defense against soil-borne pathogens. In subgroup VI, two genes were predominantly expressed in seeds, and another two genes were expressed in both seeds and siliques, supporting their putative functions in seed and silique formation.

Integrated analysis showed strong consistency between promoter element characteristics and tissue expression patterns. Genes containing seed-specific regulatory elements exhibited relatively higher expression in seed tissues. In addition, subgroup IV genes with almost no detectable expression across all examined tissues generally contained fewer types of promoter cis-elements, suggesting that these genes may not participate in regular plant growth and developmental processes. Collectively, these findings indicate that *BnaRLCK VII* genes extensively modulate the growth and development of *B. napus*, which is consistent with the functional conservation of RLCK VII family members in other plant species [14].

Transcriptome analysis revealed significant transcriptional changes in *BnaRLCK VII* genes upon *S. sclerotiorum* infection, including 27 upregulated and four downregulated genes, indicating their potential association with rapeseed disease resistance (Figure 6). Further promoter analysis suggested that these pathogen-responsive *BnaRLCK VII* genes are mainly modulated by MeJA, ABA, and SA signaling pathways (Figure 4). These three phytohormones have been well established as core regulators of *B. napus* defense against *S. sclerotiorum*. Additionally, numerous studies have confirmed that RLCK VII members in various plant species mediate hormone signaling to activate plant immune responses [71,72,73,74,75,76,77,78,79]. Therefore, we propose that *BnaRLCK VII* genes positively regulate rapeseed resistance to *S. sclerotiorum* via participation in hormone-mediated defense pathways [80].

A large number of previous studies have reported various defense-related gene families in *B. napus* that respond to *S. sclerotiorum* infection, such as receptor-like kinases, transcription factors and antioxidant enzyme families. Similar to these well-characterized resistance gene families, the *BnaRLCK VII* subfamily displays dramatic expression reprogramming under fungal stress, further verifying its essential role in rapeseed immune defense. As core cytoplasmic kinases downstream of plant pattern recognition receptors, *BnaRLCK VII* proteins can be rapidly activated after perceiving pathogen-associated molecular patterns released by *S. sclerotiorum*. Activated kinases sequentially trigger reactive oxygen species burst and calcium influx, and further initiate the MAPK cascade signaling pathway. This upstream immune signal subsequently converges with SA-, JA- and ABA-dependent signaling networks, jointly strengthening cell wall structure and limiting the expansion of fungal lesions. Moreover, the obvious functional differentiation among *BnaRLCK VII* members is also a common feature of defense gene families in rapeseed, which enables plants to build a multi-layered and stable defense system against necrotrophic pathogens.

To date, the biological functions of most *BnaRLCK VII* members remain uncharacterized in rapeseed. In this study, we selected *BnaBIK1* to explore its role in *B. napus* resistance to *S. sclerotiorum*. Tobacco transient transformation and pathogen inoculation assays showed that the lesion area was significantly reduced in *BnaBIK1*-overexpressing leaves at 24 h post inoculation, demonstrating that *BnaBIK1* enhances plant resistance against *S. sclerotiorum* (Figure 7b). Several reported RLCK VII members such as RIPK confer broad-spectrum resistance to diverse plant pathogens [81], and some RLCK VIIs are directly targeted by the same effector [13,82]. Accordingly, beyond *BnaBIK1*, other uncharacterized *BnaRLCK VII* members are also speculated to participate in *B. napus* defense against *S. sclerotiorum*, which provides promising candidate genes for future functional exploration [7,83,84].

## 5. Conclusions

Collectively, these phenotypic and transcriptomic results demonstrate that *BnaRLCK VII* family genes, particularly *BnaBIK1*, positively regulate the defense response of *B. napus* upon *S. sclerotiorum* infection. Both the induced expression patterns under pathogen stress and the transient overexpression assays in tobacco leaves confirmed that *BnaBIK1* contributes to the enhancement of plant defensive capability against fungal invasion, indicating that *BnaRLCK VII* members serve as crucial functional regulators in plant immune responses to *S. sclerotiorum*.

## Figures and Tables

**Table 1:** The identification, chromosome mapping, number of exons, physicochemical properties, and subcellular localization of 173 *BnaRLCK VII* genes.

Gene ID	Chromosome	Start	End	Gene (bp)	CDS (bp)	Exon	AA	MW (kDa)	II	AI	pI	GRAVY	Subcellular Localization
*BnaA01G0028500ZS*	A01	1600613	1603727	3115	1218	6	405	44.49	42.84	80.42	9.5	−0.291	Cell membrane
*BnaA01G0280300ZS*	A01	26098067	26100292	2226	993	3	330	36.87	52.85	82.12	9.52	−0.433	Cell membrane
*BnaA01G0283700ZS*	A01	26490604	26492524	1921	1182	5	393	43.84	31.14	78.4	5.63	−0.318	Cell membrane
*BnaA01G0314100ZS*	A01	29266349	29268529	2181	1161	6	386	43.67	39.77	78.78	6.49	−0.466	Cell membrane
*BnaA01G0383500ZS*	A01	34645682	34647896	2215	1287	4	428	47.79	35.1	77.83	9.43	−0.514	Cell membrane
*BnaA01G0387400ZS*	A01	34863404	34865396	1993	1308	5	435	48.10	37.96	78.51	6.62	−0.521	Cell membrane
*BnaA02G0007300ZS*	A02	726039	728252	2214	1167	7	388	43.38	36.54	83.45	9.35	−0.4	Cell membrane
*BnaA02G0009300ZS*	A02	803627	805471	1845	1131	5	376	41.39	41.39	80.66	9.05	−0.309	Cell membrane
*BnaA02G0011900ZS*	A02	914868	916528	1661	1116	2	371	41.84	44.72	81.37	9.5	−0.49	Cytoplasm
*BnaA02G0123000ZS*	A02	6642430	6644821	2392	696	5	231	25.93	21.1	94.55	8.32	−0.348	Cell membrane
*BnaA02G0200900ZS*	A02	12603599	12605597	1999	1344	5	447	50.44	32.22	84.56	9.06	−0.424	Cell membrane
*BnaA02G0221200ZS*	A02	13975038	13978482	3445	1371	6	456	50.99	37.71	75.88	9.7	−0.594	Cell membrane
*BnaA02G0272300ZS*	A02	18889050	18891309	2260	1419	5	472	52.71	37.76	78.5	8.77	−0.453	Cell membrane
*BnaA02G0304700ZS*	A02	27508897	27510424	1528	1140	4	379	43.18	47.44	78.18	9.34	−0.667	Cell membrane
*BnaA02G0324800ZS*	A02	29333956	29336097	2142	1206	4	401	43.06	44.05	73.89	9.9	−0.539	Cell membrane
*BnaA02G0340000ZS*	A02	30525174	30527169	1996	1341	4	446	49.86	39.86	87.47	8.93	−0.302	Cell membrane
*BnaA02G0348900ZS*	A02	31103063	31105744	2682	1413	6	470	52.07	37.93	78.19	9.54	−0.469	Cell membrane
*BnaA03G0052100ZS*	A03	2480974	2483065	2092	1254	6	417	46.40	33.19	77.46	6.35	−0.477	Cell membrane
*BnaA03G0061200ZS*	A03	2907865	2910340	2476	1515	6	504	55.77	40.31	67.72	9.12	−0.618	Cell membrane
*BnaA03G0070000ZS*	A03	3382372	3384780	2409	1731	5	576	63.67	51.84	61.13	4.54	−0.872	Cytoplasm|Cell membrane
*BnaA03G0078800ZS*	A03	3847909	3850943	3035	1554	5	517	56.29	41.43	68.55	8.96	−0.615	Cell membrane
*BnaA03G0193300ZS*	A03	10038657	10040432	1776	1182	6	393	43.83	35.28	83.84	9.18	−0.388	Cell membrane
*BnaA03G0210000ZS*	A03	10967292	10969461	2170	1098	5	365	40.35	45.28	80.44	9.39	−0.364	Cell membrane
*BnaA03G0230400ZS*	A03	11942809	11944881	2073	1203	6	400	44.63	39.83	80.4	9.08	−0.424	Cell membrane
*BnaA03G0231200ZS*	A03	11995460	11997192	1733	1296	5	431	47.83	33.36	76.22	6.25	−0.465	Cell membrane
*BnaA03G0289700ZS*	A03	15327178	15329678	2501	1707	5	568	63.44	40.38	65.02	4.77	−0.867	Cell membrane
*BnaA03G0306900ZS*	A03	16246774	16248685	1912	906	5	301	32.92	44.59	65.22	5.89	−0.662	Cell membrane
*BnaA03G0363400ZS*	A03	19330323	19331774	1452	1119	5	372	41.83	37.44	77.31	7.55	−0.39	Cell membrane
*BnaA03G0455200ZS*	A03	24800949	24803150	2202	699	4	232	26.00	27.53	86.59	6.83	−0.35	Cell membrane
*BnaA03G0550800ZS*	A03	31262531	31264796	2266	1308	6	435	47.73	44.28	78.94	9.52	−0.378	Cell membrane
*BnaA03G0551400ZS*	A03	31326519	31328706	2188	1242	5	413	45.55	38.18	85.4	9	−0.259	Cell membrane
*BnaA03G0582600ZS*	A03	42137189	42139434	2246	1275	5	424	47.11	45.68	84.17	9.3	−0.325	Cell membrane
*BnaA03G0585800ZS*	A03	42872785	42874414	1630	1167	5	388	43.45	31.12	78.63	5.89	−0.29	Cell membrane
*BnaA04G0078200ZS*	A04	6459315	6460982	1668	1155	5	384	42.63	27.76	87.32	5.71	−0.294	Cell membrane
*BnaA04G0088900ZS*	A04	10679242	10680854	1613	1353	4	450	50.28	37.3	84.49	9.08	−0.437	Cell membrane
*BnaA04G0173500ZS*	A04	18033530	18036104	2575	1014	3	337	38.62	40.15	82.7	9.61	−0.529	Cell membrane
*BnaA04G0187000ZS*	A04	19028697	19030930	2234	1257	6	418	46.33	44.24	80.96	9.23	−0.385	Cell membrane
*BnaA04G0249900ZS*	A04	22896802	22898209	1408	1341	2	446	50.30	37.16	91.12	9.23	−0.251	Cell membrane
*BnaA05G0007600ZS*	A05	481791	483825	2035	1203	8	400	43.58	34.98	80.78	7.69	−0.356	Cell membrane
*BnaA05G0067900ZS*	A05	3777462	3779468	2007	1311	4	436	48.91	34.24	88.3	9.46	−0.255	Cell membrane
*BnaA05G0352500ZS*	A05	35471031	35473387	2357	1251	5	416	46.68	34.8	87.86	5.49	−0.307	Mitochondrion
*BnaA05G0439500ZS*	A05	41238470	41240540	2071	1161	3	386	43.29	41.05	75.96	9.52	−0.516	Cell membrane
*BnaA05G0461500ZS*	A05	42302259	42308611	6353	3801	19	1266	142.14	46.52	88.71	5.94	−0.319	Cell membrane
*BnaA05G0490800ZS*	A05	44356884	44358989	2106	1656	5	551	61.48	40.96	69.49	4.76	−0.76	Cell membrane
*BnaA05G0492000ZS*	A05	44409889	44411994	2106	1656	5	551	61.48	40.96	69.49	4.76	−0.76	Cell membrane
*BnaA05G0495100ZS*	A05	44946245	44948885	2641	1467	6	488	54.12	44.59	76.91	9.36	−0.494	Cell membrane
*BnaA06G0045800ZS*	A06	3005003	3006982	1980	1200	6	399	44.35	38.45	75.46	8.6	−0.536	Cytoplasm|Nucleus
*BnaA06G0093500ZS*	A06	5618485	5620485	2001	1167	7	388	42.67	26.81	80.49	8.57	−0.264	Cell membrane
*BnaA06G0093600ZS*	A06	5625317	5626987	1671	1263	6	420	45.40	33.72	82.24	9.34	−0.275	Cell membrane
*BnaA06G0145100ZS*	A06	8712796	8713742	947	774	3	257	28.77	34.58	92.96	6.5	−0.148	Cell membrane
*BnaA06G0386800ZS*	A06	44798128	44800022	1895	1335	5	444	49.85	49.53	76.87	6.53	−0.497	Cell membrane
*BnaA06G0418700ZS*	A06	46772988	46774997	2010	1071	4	356	40.28	42.55	79.94	9.14	−0.663	Cell membrane
*BnaA07G0030800ZS*	A07	2651288	2653705	2418	1233	6	410	45.16	42.71	78.27	9.42	−0.391	Cell membrane
*BnaA07G0055600ZS*	A07	10380678	10382344	1667	1374	4	457	50.98	38.03	75.75	9.36	−0.578	Cell membrane
*BnaA07G0131700ZS*	A07	17110224	17111573	1350	1095	4	364	40.84	41.61	87.83	8.62	−0.18	Cell membrane
*BnaA07G0165300ZS*	A07	19430516	19432434	1919	1479	4	492	54.97	38.55	85.73	8.71	−0.291	Cell membrane
*BnaA07G0243700ZS*	A07	23815420	23817135	1716	1146	6	381	42.25	32.07	82.36	9.01	−0.28	Cell membrane
*BnaA07G0243800ZS*	A07	23820838	23824312	3475	1344	6	447	49.66	40.19	82.21	9.9	−0.453	Cell membrane
*BnaA07G0251500ZS*	A07	24289509	24291527	2019	1188	5	395	44.34	33.15	78.73	9.11	−0.458	Cell membrane
*BnaA07G0333900ZS*	A07	29364526	29366208	1683	1356	5	451	50.71	31.33	84.55	9.24	−0.407	Cell membrane
*BnaA07G0360100ZS*	A07	30745573	30751092	5520	1065	5	354	39.58	34.05	76.78	9.7	−0.618	Cell membrane
*BnaA07G0360200ZS*	A07	30753087	30754966	1880	1152	6	383	42.48	33.14	78.15	9.37	−0.368	Cell membrane
*BnaA08G0106100ZS*	A08	16518062	16519536	1475	1191	4	396	45.16	38.27	80.45	9.54	−0.555	Cell membrane
*BnaA08G0179300ZS*	A08	21365828	21367947	2120	1104	5	367	40.70	30.63	88.15	7.79	−0.202	Cell membrane
*BnaA08G0179400ZS*	A08	21369651	21370408	758	483	2	160	18.01	51.97	71.19	9.71	−0.543	Nucleus
*BnaA08G0179500ZS*	A08	21372098	21374525	2428	711	5	236	25.77	41.03	81.44	8.87	−0.192	Cell membrane
*BnaA08G0232800ZS*	A08	24234663	24237522	2860	1233	5	410	45.08	37.33	79.2	9.62	−0.376	Cell membrane
*BnaA08G0246800ZS*	A08	25009886	25011440	1555	1209	5	402	44.85	35.16	85.1	8.73	−0.251	Cell membrane
*BnaA08G0301500ZS*	A08	27502984	27504934	1951	1254	5	417	46.42	31.54	77.6	8.48	−0.544	Cell membrane
*BnaA09G0110900ZS*	A09	6479911	6482342	2432	1239	6	412	45.45	40.31	78.62	9.43	−0.407	Cell membrane
*BnaA09G0126800ZS*	A09	7600315	7601849	1535	1134	5	377	41.91	34.87	87.67	5.53	−0.318	Cell membrane
*BnaA09G0211800ZS*	A09	14910300	14912748	2449	864	3	287	32.66	36.88	81.46	9.29	−0.477	Cell membrane
*BnaA09G0259000ZS*	A09	19289228	19291400	2173	1368	5	455	50.54	34.71	80.55	8.84	−0.427	Cell membrane
*BnaA09G0448600ZS*	A09	50382986	50384761	1776	1128	7	375	42.48	38.48	87.87	9.32	−0.321	Cell membrane
*BnaA09G0633800ZS*	A09	61338963	61341145	2183	624	3	207	21.96	46.73	65.51	9.87	−0.606	Cell membrane
*BnaA09G0668900ZS*	A09	63151137	63153608	2472	1272	5	423	47.21	35.54	74.21	9.02	−0.592	Cell membrane
*BnaA09G0670100ZS*	A09	63196887	63199007	2121	513	2	170	19.08	58.47	86	10.17	−0.405	Cytoplasm|Nucleus|Cell membrane
*BnaA10G0055400ZS*	A10	3447415	3449482	2068	1227	6	408	45.27	41.53	83.14	9.46	−0.347	Cell membrane
*BnaA10G0123900ZS*	A10	16995712	16997968	2257	1266	6	421	47.59	36.98	88.17	6.59	−0.434	Cell membrane
*BnaA10G0186500ZS*	A10	21091771	21094648	2878	1551	5	516	56.02	38.13	69.07	9.13	−0.602	Cell membrane
*BnaA10G0201400ZS*	A10	21897813	21899995	2183	1779	5	592	66.80	63.02	61.59	4.67	−0.912	Cell membrane
*BnaA10G0224300ZS*	A10	22965865	22968015	2151	1368	5	455	50.36	35.35	75.05	6.3	−0.563	Cell membrane
*BnaA10G0291700ZS*	A10	26098055	26100067	2013	1227	3	408	45.84	41.46	82.13	9.41	−0.491	Cell membrane
*BnaC01G0033400ZS*	C01	1879645	1882151	2507	1293	6	430	47.40	44.96	81.63	9.54	−0.283	Cell membrane
*BnaC01G0170200ZS*	C01	12419961	12433868	13,908	1161	5	386	43.72	39.11	78.78	6.69	−0.473	Cell membrane
*BnaC01G0343300ZS*	C01	39104531	39107524	2994	1254	5	417	46.15	46.16	83.74	9.35	−0.335	Cell membrane
*BnaC01G0347000ZS*	C01	39739208	39741153	1946	1182	5	393	43.76	29.12	78.65	5.75	−0.305	Cell membrane
*BnaC01G0387700ZS*	C01	44969973	44972232	2260	1158	5	385	43.50	40.8	80	6.13	−0.441	Cell membrane
*BnaC01G0479800ZS*	C01	54341272	54344870	3599	1446	5	481	53.77	36.04	78.57	9.38	−0.47	Cell membrane
*BnaC01G0491300ZS*	C01	56268070	56270022	1953	1308	5	435	48.23	39.01	77.59	6.17	−0.548	Cell membrane
*BnaC01G0495800ZS*	C01	56547612	56549812	2201	858	5	285	31.74	39.83	79.61	9.41	−0.507	Cell membrane
*BnaC02G0004800ZS*	C02	408638	410878	2241	1167	7	388	43.37	35.65	84.46	9.27	−0.404	Cell membrane
*BnaC02G0006900ZS*	C02	521451	523280	1830	1128	5	375	41.34	38.43	80.4	9.12	−0.331	Cytoplasm
*BnaC02G0008200ZS*	C02	573409	575518	2110	1230	4	409	46.06	43.89	83.35	9.35	−0.438	Cell membrane
*BnaC02G0053400ZS*	C02	3350863	3353263	2401	1359	4	452	49.50	29.24	75.38	6.11	−0.479	Cell membrane
*BnaC02G0152500ZS*	C02	11449520	11451715	2196	1248	6	415	46.70	29.12	87.83	6.78	−0.401	Cell membrane
*BnaC02G0206600ZS*	C02	17686030	17687747	1718	729	5	242	27.55	29.08	78.97	6.6	−0.418	Cell membrane
*BnaC02G0242800ZS*	C02	22323726	22337012	13,287	783	6	260	29.01	38.02	76.88	9.82	−0.381	Cell membrane
*BnaC02G0268000ZS*	C02	25540276	25542229	1954	1344	5	447	50.55	32.88	85.64	9.08	−0.408	Cell membrane
*BnaC02G0297700ZS*	C02	28489509	28492765	3257	1098	4	365	41.01	45.5	73.12	9.87	−0.718	Cell membrane
*BnaC02G0370300ZS*	C02	39631887	39633893	2007	1419	5	472	52.67	37.21	79.32	8.73	−0.458	Cell membrane
*BnaC02G0414900ZS*	C02	51378807	51380531	1725	1224	4	407	45.94	46.86	78.99	9.24	−0.611	Cell membrane
*BnaC02G0437900ZS*	C02	53962699	53964799	2101	1158	4	385	41.58	43.2	75.69	9.9	−0.513	Cell membrane
*BnaC02G0457100ZS*	C02	56490653	56492599	1947	1227	5	408	45.70	53.98	92.5	9.17	−0.302	Cell membrane
*BnaC02G0470100ZS*	C02	57566426	57569485	3060	1425	6	474	52.46	39.14	77.95	9.55	−0.468	Cell membrane
*BnaC03G0060000ZS*	C03	3115468	3117843	2376	1473	6	490	54.82	35.04	80.61	9.1	−0.405	Cell membrane
*BnaC03G0079200ZS*	C03	4105784	4108215	2432	1740	5	579	64.05	51.47	61.99	4.48	−0.867	Cell membrane
*BnaC03G0090000ZS*	C03	4750475	4753524	3050	1551	5	516	55.89	40.87	68.68	8.96	−0.601	Cell membrane
*BnaC03G0247500ZS*	C03	15196880	15199261	2382	1215	5	404	44.84	42.41	79.93	9.22	−0.365	Cell membrane
*BnaC03G0271800ZS*	C03	16866115	16868173	2059	1290	5	429	47.47	37.42	83.15	9.06	−0.301	Cell membrane
*BnaC03G0272800ZS*	C03	16992590	16994508	1919	1296	5	431	48.05	32.59	76.45	6.19	−0.459	Cell membrane
*BnaC03G0347900ZS*	C03	23585840	23588339	2500	1710	5	569	63.58	41.16	64.9	4.73	−0.875	Cell membrane
*BnaC03G0368200ZS*	C03	24817331	24819243	1913	1329	5	442	48.77	36.21	73.1	5.69	−0.574	Nucleus
*BnaC03G0443500ZS*	C03	31046617	31048072	1456	1119	5	372	41.87	37.56	76.53	7	−0.393	Cell membrane
*BnaC03G0699000ZS*	C03	67450913	67453956	3044	978	6	325	36.54	40.48	82.49	9.75	−0.357	Cell membrane
*BnaC04G0071800ZS*	C04	6252159	6253731	1573	1161	6	386	42.93	34.39	85.13	8.92	−0.342	Cell membrane
*BnaC04G0076600ZS*	C04	6763141	6765100	1960	1308	4	435	48.84	38.45	88.51	9.5	−0.269	Cell membrane
*BnaC04G0190800ZS*	C04	18211510	18213521	2012	1548	4	515	57.67	35.55	84.16	8.39	−0.234	Cell membrane
*BnaC04G0190900ZS*	C04	18214195	18216091	1897	1278	5	425	47.33	38	85.34	9.21	−0.296	Cell membrane
*BnaC04G0235600ZS*	C04	32288854	32291106	2253	1143	5	380	42.34	46.69	84.68	9.1	−0.265	Cell membrane
*BnaC04G0238600ZS*	C04	33071229	33072808	1580	1194	5	397	44.21	31.53	78.82	5.74	−0.305	Cell membrane
*BnaC04G0364300ZS*	C04	48611511	48613185	1675	1155	5	384	42.66	30.76	88.33	6.04	−0.291	Cell membrane
*BnaC04G0369100ZS*	C04	49130782	49132388	1607	1353	4	450	50.25	36.87	85.13	9.15	−0.42	Cell membrane
*BnaC04G0471500ZS*	C04	59813742	59817161	3420	1275	5	424	48.15	34.52	82.1	9.5	−0.458	Cell membrane
*BnaC04G0489300ZS*	C04	61565462	61567749	2288	1257	6	418	46.34	45.23	80.02	9.23	−0.389	Cell membrane
*BnaC04G0564900ZS*	C04	67821158	67822751	1594	1314	4	437	49.13	42.88	84.97	9.31	−0.38	Cell membrane
*BnaC05G0054900ZS*	C05	3112105	3113624	1520	747	4	248	27.96	47.57	87.62	9.7	−0.375	Cytoplasm
*BnaC05G0055100ZS*	C05	3136692	3138612	1921	1227	6	408	45.28	40.96	81.94	9.49	−0.37	Cell membrane
*BnaC05G0057700ZS*	C05	3254111	3256026	1916	1275	5	424	46.95	35.84	75.85	8.88	−0.545	Cell membrane
*BnaC05G0114700ZS*	C05	7111714	7117739	6026	1056	7	351	38.71	25	86.47	9.27	−0.216	Cell membrane
*BnaC05G0114800ZS*	C05	7119582	7121378	1797	1266	6	421	45.58	32.96	82.04	9.48	−0.278	Cell membrane
*BnaC05G0176200ZS*	C05	11807341	11809013	1673	1131	6	376	41.47	30.69	89.41	8.86	−0.18	Cell membrane
*BnaC05G0210400ZS*	C05	15306398	15308179	1782	1128	7	375	42.50	38.65	87.87	9.36	−0.322	Cytoplasm|Nucleus|Cell membrane
*BnaC05G0500500ZS*	C05	53953039	53957479	4441	1281	4	426	47.89	38.65	77.98	9.43	−0.492	Cell membrane
*BnaC05G0521100ZS*	C05	54940579	54942412	1834	1326	5	441	48.79	42.09	75.9	5.78	−0.541	Cell membrane
*BnaC05G0561500ZS*	C05	57389570	57391693	2124	1194	4	397	43.63	46.71	67.76	9.39	−0.608	Cell membrane
*BnaC06G0267100ZS*	C06	37422997	37425515	2519	1146	6	381	42.20	31.76	81.34	8.84	−0.291	Cell membrane
*BnaC06G0267300ZS*	C06	37459919	37463002	3084	1320	6	439	48.76	39.71	80.39	9.9	−0.49	Cell membrane
*BnaC06G0277300ZS*	C06	38308934	38310913	1980	1188	5	395	44.35	33.88	80.71	9.13	−0.439	Cell membrane
*BnaC06G0288700ZS*	C06	39391240	39392681	1442	1191	4	396	44.52	29.49	90.1	9.07	−0.296	Cell membrane
*BnaC06G0363600ZS*	C06	46454950	46456741	1792	1185	5	394	43.51	41.67	82.16	9.77	−0.323	Cell membrane
*BnaC06G0392100ZS*	C06	48554517	48556220	1704	1362	5	453	50.88	31.01	84.37	9.28	−0.4	Cell membrane
*BnaC06G0423800ZS*	C06	50406383	50411199	4817	3540	8	1179	129.82	36.78	104.68	6.36	−0.056	Cytoplasm|Cell membrane
*BnaC07G0020800ZS*	C07	3975043	3976078	1036	825	2	274	29.88	42.71	84.42	5.01	−0.134	Cell membrane
*BnaC07G0056000ZS*	C07	9226872	9229295	2424	1233	6	410	45.16	43.97	78.02	9.42	−0.395	Cell membrane
*BnaC07G0081700ZS*	C07	17854226	17855907	1682	1374	4	457	51.02	37.76	76.39	9.31	−0.572	Cell membrane
*BnaC07G0109400ZS*	C07	22191664	22195220	3557	1131	5	376	41.85	45.21	84.28	6.03	−0.392	Cell membrane
*BnaC07G0193900ZS*	C07	32751645	32752910	1266	888	5	295	32.92	34.87	86.54	8.39	−0.158	Cell membrane
*BnaC07G0256700ZS*	C07	40333245	40334939	1695	1209	4	402	45.51	42.17	80.5	9.35	−0.616	Cytoplasm
*BnaC07G0280000ZS*	C07	42399519	42401504	1986	1371	6	456	49.52	37.37	77.39	9.8	−0.446	Cell membrane
*BnaC07G0301300ZS*	C07	44515174	44517045	1872	1329	5	442	49.41	43.22	77.67	6.26	−0.469	Cell membrane
*BnaC07G0527400ZS*	C07	59588193	59590450	2258	1308	6	435	47.75	46.49	78.71	9.48	−0.376	Cell membrane
*BnaC07G0527500ZS*	C07	59591192	59593538	2347	1179	6	392	42.99	43.12	80.54	9.23	−0.335	Cell membrane
*BnaC08G0032500ZS*	C08	2448102	2449912	1811	1269	5	422	47.10	33.19	78.06	8.62	−0.544	Cell membrane
*BnaC08G0154100ZS*	C08	26179259	26180741	1483	1191	4	396	44.97	40.76	82.4	9.54	−0.546	Cell membrane
*BnaC08G0261000ZS*	C08	34949165	34950730	1566	1137	6	378	41.90	38.06	85.61	8.75	−0.281	Cell membrane
*BnaC08G0282100ZS*	C08	36732109	36734142	2034	1197	5	398	44.06	40.8	79.37	9.62	−0.419	Cell membrane
*BnaC08G0491500ZS*	C08	51090513	51092689	2177	1290	6	429	46.21	34.64	76.88	9.59	−0.391	Cell membrane
*BnaC08G0534000ZS*	C08	53602662	53605228	2567	1332	5	443	49.48	36.04	72.19	9.17	−0.618	Cell membrane
*BnaC09G0027400ZS*	C09	1823619	1829253	5635	2742	17	913	101.18	43.8	82.19	6.24	−0.311	Cell membrane
*BnaC09G0113200ZS*	C09	7938779	7940795	2017	1251	6	416	45.87	41.54	77.62	9.44	−0.41	Cell membrane
*BnaC09G0133200ZS*	C09	9772991	9774527	1537	1137	5	378	41.99	34.32	86.4	5.44	−0.347	Cell membrane
*BnaC09G0245900ZS*	C09	22892126	22893978	1853	1233	4	410	46.15	39.79	81.29	9.69	−0.586	Cytoplasm
*BnaC09G0305200ZS*	C09	30261920	30263691	1772	1323	6	440	48.78	35.15	78.2	8.75	−0.468	Cell membrane
*BnaC09G0395900ZS*	C09	50500570	50502775	2206	1152	6	383	43.39	36.4	87.26	7.12	−0.461	Cell membrane
*BnaC09G0478200ZS*	C09	58857797	58860665	2869	1557	5	518	56.17	38.02	68.8	9.13	−0.602	Cell membrane
*BnaC09G0499700ZS*	C09	60687422	60690059	2638	1761	5	586	66.06	62.76	63.21	4.75	−0.889	Cell membrane
*BnaC09G0512600ZS*	C09	61537685	61546846	9162	3888	33	1295	145.96	47.67	79.09	5.97	−0.531	Cytoplasm
*BnaC09G0529300ZS*	C09	62667147	62669317	2171	1368	5	455	50.33	34.93	75.05	6.3	−0.562	Cell membrane
*BnaC09G0611200ZS*	C09	67529110	67530511	1402	1248	3	415	46.67	41.67	82.17	9.44	−0.513	Cell membrane
*BnaC09G0618400ZS*	C09	68017340	68019038	1699	1173	6	390	43.37	36.51	83.77	9.21	−0.394	Cell membrane
*Bnascaffold0025G0039100ZS*	scaffold0025	3830877	3833362	2486	1128	7	375	42.48	38.48	87.87	9.32	−0.321	Cell membrane
*Bnascaffold0630G0000100ZS*	scaffold0630	1	988	988	582	4	193	21.88	29.88	94.92	5.2	−0.211	Cytoplasm

## Data Availability

The original contributions presented in this study are included in the article/Supplementary Materials. Further inquiries can be directed to the corresponding author.

## Supplementary Materials

The following supporting information can be downloaded at https://www.mdpi.com/article/10.3390/genes17070790/s1, Figure S1: The numbers of different types *cis*-acting elements in the promoters of 172 *BnaRLCK VII* genes; Table S1: The signal peptide analysis based on the protein sequences of 173 *BnaRLCK* VIIs; Table S2: The duplication of *BnaRLCK VIIs*; Table S3: The selective pressure information of 173 *BnaRLCK VII* genes; Table S4: The primer list. All the primers used in this study are listed.

## Abbreviations

The following abbreviations are used in this manuscript:

RLCKs	Receptor-like cytoplasmic kinases
DEGs	Differentially expressed genes
RLKs	Receptor-like kinases
KD	Kinase domains
TM	Transmembrane domain
ECD	Extracellular ligand-binding domain
LRR	Leucine-rich repeat
WAK	Wall-associated kinase
PRRs	Pattern recognition receptors
PAMP	Pathogen-associated molecular patterns
PTI	PAMP-triggered immunity
ETI	Effector-triggered immunity
ROS	Reactive oxygen species
BSK1	BR-SIGNALING KINASE1
FLS2	FLAGELLIN SENSING2
BIK1	BOTRYTIS-INDUCED KINASE1
PBS1	AvrPphB-susceptible 1
RPS5	Resistance to *Pseudomonas syringae* 5
PBL	PBS1-like
MAPK	Mitogen-activated protein kinase
MAPKKK5	Mitogen-activated protein kinase kinase kinase 5
xopAC	Xanthomonas outer protein AC
AvrAC	AvrBs3-like effector AC
BSK	Brassinosteroid-Signaling Kinase
CDD	Conserved Domain Database
GRAVY	Grand Average of Hydropathicity
MW	Molecular Weight
ML	Maximum Likelihood
GFF3	Generic Feature Format Version 3
MEME Suite	Multiple EM for Motif Elicitation Suite
MCScanX	Multiple Collinearity Scan toolkit X
FPKM	Fragments Per Kilobase per Million bases
RT-qPCR	Reverse Transcription quantitative real-time Polymerase Chain Reaction
ORF	Open Read Frame
PDA	Potato Dextrose Agar
PKc_like domain	Protein Kinases, catalytic-like domain
WGD	Whole-Genome Duplication
SD	Segmental Duplication
DSD	Dispersed Duplication
PD	Proximal Duplication
TD	Tandem Duplication
MeJA	Methyl Jasmonate
GA	Gibberellins
ABA	Abscisic Acid
SA	Salicylic Acid
DAF	Days After Flowering
CDS	Coding Sequence
WGT	Whole-Genome Triplication
HP_HAP_like	Histidine Phosphatase
RING_Ubox	RING-finger and U-box domain
RPA1_DBD_A_like	Replication Protein A1-DNA Binding Domain A-like
NHL	Ncl-1, HT2A and Lin-41 repeat domain
RNA-seq	RNA-Sequencing
PGN	Peptidoglycan
