# Supplementary material for: Genome-Wide Analysis of BnaRLCK VII Gene Family in Brassica napus and Investigation of Its Function in Resistance to Sclerotinia sclerotiorum
Source: Genes (Basel). 2026 Jul 12;17(7):790. doi: 10.3390/genes17070790 (PMC13410009; doi:10.3390/genes17070790)
Supplement: Supplementary file 1 [file genes-17-00790-s001.zip › Table S2.pdf]

Table S2: The duplication of *BnaRLCK VIIs*

Gene ID	Type of duplication
BnaA01T0028500ZS	WGD or Segmental
BnaA01T0280300ZS	WGD or Segmental
BnaA01T0283700ZS	WGD or Segmental
BnaA01T0314100ZS	WGD or Segmental
BnaA01T0383500ZS	WGD or Segmental
BnaA01T0387400ZS	WGD or Segmental
BnaA02T0007300ZS	WGD or Segmental
BnaA02T0009300ZS	WGD or Segmental
BnaA02T0011900ZS	WGD or Segmental
BnaA02T0123000ZS	WGD or Segmental
BnaA02T0200900ZS	WGD or Segmental
BnaA02T0221200ZS	WGD or Segmental
BnaA02T0272300ZS	Tandem
BnaA02T0304700ZS	WGD or Segmental
BnaA02T0324800ZS	WGD or Segmental
BnaA02T0340000ZS	WGD or Segmental
BnaA02T0348900ZS	WGD or Segmental
BnaA03T0052100ZS	WGD or Segmental
BnaA03T0061200ZS	WGD or Segmental
BnaA03T0070000ZS	WGD or Segmental
BnaA03T0078800ZS	WGD or Segmental
BnaA03T0193300ZS	WGD or Segmental
BnaA03T0210000ZS	WGD or Segmental
BnaA03T0230400ZS	WGD or Segmental
BnaA03T0231200ZS	WGD or Segmental
BnaA03T0289700ZS	WGD or Segmental
BnaA03T0306900ZS	WGD or Segmental
BnaA03T0363400ZS	WGD or Segmental
BnaA03T0455200ZS	Dispersed
BnaA03T0550800ZS	WGD or Segmental
BnaA03T0551400ZS	Proximal

Gene ID	Type of duplication
BnaA01T0028500ZS	WGD or Segmental
BnaA03T0582600ZS	WGD or Segmental
BnaA03T0585800ZS	WGD or Segmental
BnaA04T0078200ZS	WGD or Segmental
BnaA04T0088900ZS	WGD or Segmental
BnaA04T0173500ZS	WGD or Segmental
BnaA04T0187000ZS	WGD or Segmental
BnaA04T0249900ZS	WGD or Segmental
BnaA05T0007600ZS	WGD or Segmental
BnaA05T0067900ZS	WGD or Segmental
BnaA05T0352500ZS	WGD or Segmental
BnaA05T0439500ZS	WGD or Segmental
BnaA05T0461500ZS	WGD or Segmental
BnaA05T0490800ZS	WGD or Segmental
BnaA05T0492000ZS	WGD or Segmental
BnaA05T0495100ZS	Dispersed
BnaA06T0045800ZS	WGD or Segmental
BnaA06T0093500ZS	WGD or Segmental
BnaA06T0093600ZS	WGD or Segmental
BnaA06T0145100ZS	WGD or Segmental
BnaA06T0386800ZS	WGD or Segmental
BnaA06T0418700ZS	WGD or Segmental
BnaA07T0030800ZS	WGD or Segmental
BnaA07T0055600ZS	WGD or Segmental
BnaA07T0131700ZS	WGD or Segmental
BnaA07T0165300ZS	WGD or Segmental
BnaA07T0243700ZS	WGD or Segmental
BnaA07T0243800ZS	WGD or Segmental
BnaA07T0251500ZS	WGD or Segmental
BnaA07T0333900ZS	WGD or Segmental
BnaA07T0360100ZS	WGD or Segmental
BnaA07T0360200ZS	WGD or Segmental

Gene ID	Type of duplication
BnaA01T0028500ZS	WGD or Segmental
BnaA08T0106100ZS	WGD or Segmental
BnaA08T0179300ZS	WGD or Segmental
BnaA08T0179400ZS	WGD or Segmental
BnaA08T0179500ZS	Dispersed
BnaA08T0232800ZS	WGD or Segmental
BnaA08T0246800ZS	WGD or Segmental
BnaA08T0301500ZS	WGD or Segmental
BnaA09T0110900ZS	WGD or Segmental
BnaA09T0126800ZS	WGD or Segmental
BnaA09T0211800ZS	WGD or Segmental
BnaA09T0259000ZS	WGD or Segmental
BnaA09T0448600ZS	WGD or Segmental
BnaA09T0633800ZS	WGD or Segmental
BnaA09T0668900ZS	WGD or Segmental
BnaA09T0670100ZS	WGD or Segmental
BnaA10T0055400ZS	Dispersed
BnaA10T0123900ZS	WGD or Segmental
BnaA10T0186500ZS	WGD or Segmental
BnaA10T0201400ZS	WGD or Segmental
BnaA10T0224300ZS	WGD or Segmental
BnaA10T0291700ZS	WGD or Segmental
BnaC01T0033400ZS	WGD or Segmental
BnaC01T0170200ZS	WGD or Segmental
BnaC01T0343300ZS	WGD or Segmental
BnaC01T0347000ZS	WGD or Segmental
BnaC01T0387700ZS	WGD or Segmental
BnaC01T0479800ZS	WGD or Segmental
BnaC01T0491300ZS	WGD or Segmental
BnaC01T0495800ZS	WGD or Segmental
BnaC02T0004800ZS	WGD or Segmental
BnaC02T0006900ZS	WGD or Segmental

Gene ID	Type of duplication
BnaA01T0028500ZS	WGD or Segmental
BnaC02T0008200ZS	WGD or Segmental
BnaC02T0053400ZS	WGD or Segmental
BnaC02T0152500ZS	WGD or Segmental
BnaC02T0206600ZS	Dispersed
BnaC02T0242800ZS	WGD or Segmental
BnaC02T0268000ZS	WGD or Segmental
BnaC02T0297700ZS	WGD or Segmental
BnaC02T0370300ZS	WGD or Segmental
BnaC02T0414900ZS	WGD or Segmental
BnaC02T0437900ZS	WGD or Segmental
BnaC02T0457100ZS	WGD or Segmental
BnaC02T0470100ZS	WGD or Segmental
BnaC03T0060000ZS	WGD or Segmental
BnaC03T0079200ZS	WGD or Segmental
BnaC03T0090000ZS	WGD or Segmental
BnaC03T0247500ZS	WGD or Segmental
BnaC03T0271800ZS	WGD or Segmental
BnaC03T0272800ZS	WGD or Segmental
BnaC03T0347900ZS	WGD or Segmental
BnaC03T0368200ZS	WGD or Segmental
BnaC03T0443500ZS	WGD or Segmental
BnaC03T0699000ZS	WGD or Segmental
BnaC04T0071800ZS	WGD or Segmental
BnaC04T0076600ZS	WGD or Segmental
BnaC04T0190800ZS	WGD or Segmental
BnaC04T0190900ZS	WGD or Segmental
BnaC04T0235600ZS	WGD or Segmental
BnaC04T0238600ZS	WGD or Segmental
BnaC04T0364300ZS	WGD or Segmental
BnaC04T0369100ZS	WGD or Segmental
BnaC04T0471500ZS	WGD or Segmental

Gene ID	Type of duplication
BnaA01T0028500ZS	WGD or Segmental
BnaC04T0489300ZS	WGD or Segmental
BnaC04T0564900ZS	WGD or Segmental
BnaC05T0054900ZS	WGD or Segmental
BnaC05T0055100ZS	Proximal
BnaC05T0057700ZS	WGD or Segmental
BnaC05T0114700ZS	WGD or Segmental
BnaC05T0114800ZS	WGD or Segmental
BnaC05T0176200ZS	WGD or Segmental
BnaC05T0210400ZS	WGD or Segmental
BnaC05T0500500ZS	WGD or Segmental
BnaC05T0521100ZS	WGD or Segmental
BnaC05T0561500ZS	WGD or Segmental
BnaC06T0267100ZS	WGD or Segmental
BnaC06T0267300ZS	WGD or Segmental
BnaC06T0277300ZS	WGD or Segmental
BnaC06T0288700ZS	WGD or Segmental
BnaC06T0363600ZS	WGD or Segmental
BnaC06T0392100ZS	WGD or Segmental
BnaC06T0423800ZS	WGD or Segmental
BnaC07T0020800ZS	Dispersed
BnaC07T0056000ZS	WGD or Segmental
BnaC07T0081700ZS	WGD or Segmental
BnaC07T0109400ZS	Dispersed
BnaC07T0193900ZS	WGD or Segmental
BnaC07T0256700ZS	WGD or Segmental
BnaC07T0280000ZS	WGD or Segmental
BnaC07T0301300ZS	WGD or Segmental
BnaC07T0527400ZS	WGD or Segmental
BnaC07T0527500ZS	Tandem
BnaC08T0032500ZS	WGD or Segmental
BnaC08T0154100ZS	WGD or Segmental

Gene ID	Type of duplication
BnaA01T0028500ZS	WGD or Segmental
BnaC08T0261000ZS	WGD or Segmental
BnaC08T0282100ZS	WGD or Segmental
BnaC08T0491500ZS	WGD or Segmental
BnaC08T0534000ZS	WGD or Segmental
BnaC09T0027400ZS	WGD or Segmental
BnaC09T0113200ZS	WGD or Segmental
BnaC09T0133200ZS	WGD or Segmental
BnaC09T0245900ZS	WGD or Segmental
BnaC09T0305200ZS	WGD or Segmental
BnaC09T0395900ZS	WGD or Segmental
BnaC09T0478200ZS	WGD or Segmental
BnaC09T0499700ZS	WGD or Segmental
BnaC09T0512600ZS	WGD or Segmental
BnaC09T0529300ZS	WGD or Segmental
BnaC09T0611200ZS	WGD or Segmental
BnaC09T0618400ZS	WGD or Segmental
Bnascaffold0025T0039100ZS	Dispersed
Bnascaffold0630T0000100ZS	Dispersed

Note: Tandem means that gene might position directly adjacent to the original segment; Dispersed means that the gene might arise from transposition; Proximal means that the gene might arise from small-scale transposition or arise from tandem duplication and insertion of some other genes; WGD or segmental means that the gene might arise from Whole Genome Duplication or Segmental Duplication.
