# Supplementary figures and images for: Genome-Wide Analysis of BnaRLCK VII Gene Family in Brassica napus and Investigation of Its Function in Resistance to Sclerotinia sclerotiorum
Source: Genes (Basel). 2026 Jul 12;17(7):790. doi: 10.3390/genes17070790 (PMC13410009; doi:10.3390/genes17070790)

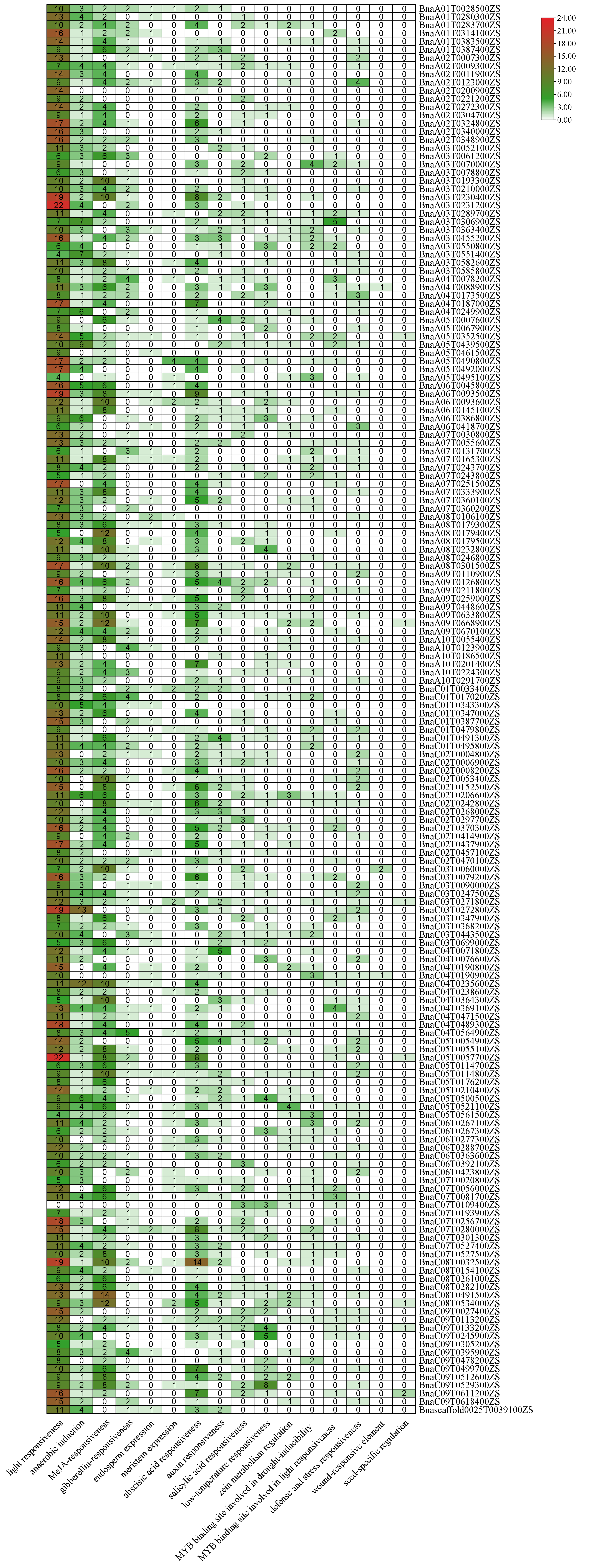

Supplement: Supplementary file 1 [file genes-17-00790-s001.zip › Figure S1.tif]
